# Cardiovascular benefits of a home-based exercise program in patients with sickle cell disease

**DOI:** 10.1371/journal.pone.0250128

**Published:** 2021-05-12

**Authors:** Jonas Alves de Araujo Junior, Daniele Andreza Antonelli Rossi, Taina Fabri Carneiro Valadão, Juliana Cristina Milan-Mattos, Aparecida Maria Catai, Tatiana de Oliveira Sato, Joao Carlos Hueb, Silmeia Garcia Zanati Bazan, Paula Oliveira Montandon Hokama, Newton Key Hokama, Meliza Goi Roscani

**Affiliations:** 1 Department of Internal Medicine, Sao Paulo State University Julio de Mesquita Filho–Unesp, Botucatu, Sao Paulo, Brazil; 2 Department of Physiotherapy, Federal University of Sao Carlos–UFSCar, São Carlos, Brazil; 3 Department of Medicine, Federal University of Sao Carlos–UFSCar, São Carlos, Brazil; Prince Sattam Bin Abdulaziz University, College of Applied Medical Sciences, SAUDI ARABIA

## Abstract

**Background:**

Physical inactivity is an important risk factor for cardiovascular disease. The benefits of exercise in patients with chronic diseases, including cardiovascular diseases, are well established. For patients with sickle cell disease, medical recommendation was to avoid physical exercise for fear of triggering painful crises or increasing the impairment of the cardiopulmonary function. Only recently, studies have shown safety in exercise programs for this population. Despite that, there is no report that assess the effects of physical exercise on cardiac parameters in patients with sickle cell disease.

**Objective:**

This study aimed to evaluate the impact of regular physical exercise (a home-based program) on cardiovascular function in patients with sickle cell disease.

**Design:**

A quasi-randomized prospective controlled trial.

**Setting:**

During the years 2015 and 2016, we started recruiting among adult patients treated at a Brazilian Center for Patients with Sickle Cell Disease to participate in a study involving a home exercise program. The experimental (exercise) and control groups were submitted to clinical evaluation and cardiovascular tests before and after the intervention. Analysis of variance was applied to compare groups, considering time and group factors.

**Participants:**

Twenty-seven adult outpatients with a sickle cell disease diagnosis.

**Interventions:**

Exercise group (N = 14): a regular home-based aerobic exercise program, three to five times per week not exceeding give times per week, for eight weeks; no prescription for the control group (N = 13).

**Main outcome measures:**

Echocardiographic and treadmill test parameters.

**Results:**

The exercise group showed significant improvement in cardiovascular tests, demonstrated by increased distance traveled on a treadmill (p<0.01), increased ejection fraction (p < 0.01) and improvement of diastolic function assessed by mitral tissue Doppler E’ wave on echocardiography (p = 0.04). None of the patients presented a sickle cell crisis or worsening of symptoms during the exercise program.

**Conclusion:**

The selected home-based exercise program is safe, feasible, and promotes a favorable impact on functional capacity and cardiovascular function in sickle cell disease patients.

## Introduction

Sickle cell disease (SCD) refers to a group of inherited hemolytic diseases attributable to hemoglobin mutation, with a hemoglobin S pattern predominant in electrophoresis [1]. SCD is the most prevalent hematological genetic disease in Brazil and affects millions of people worldwide. Brazilian Ministry of Health estimates that about 60 thousand people with the disease live in Brazil [2]. Polymerization of deoxygenated hemoglobin S molecules causes sickling of erythrocytes, which may lead to vessel occlusion and multiple organ ischemia, resulting in reduced functional capacity (FC) and cardiovascular function, frequent painful crises, chronic inflammation, organ failure, and impaired quality of life [3].

Clinical expression of sickle cell disease is very heterogeneous; the frequency and degree of complications are highly variable, as are the number of annual infections and crisis episodes, hospitalizations, transfusion requirements, in addition to the degree of renal, pulmonary, cerebral and cardiovascular impairment, and the presence or absence of malleolar ulcers. Therapeutic and genetic factors such as the proportion of fetal hemoglobin, concomitant alpha thalassemia, use of hydroxyurea, overload and iron chelation modulate the severity of the disease [4].

The beneficial effect of physical exercise on quality of life and functional capacity is well-described in patients with cardiovascular disease [5]. However, exercise activity induced metabolic changes, such as increased lactic acid production, dehydration, and body temperature changes, by increasing Hemoglobin S desoxigenation, could hypothetically unbalance the microvascular and endothelial environment in patients with sickle cell disease and produce hipoxya. As an indication of this possibility, an increase in TNF alpha and nitric oxide metabolites after exercise were demonstrated in a group of women with sickle cell disease compared to controls [6].

The first recommendations for patients with sickle cell disease to practice physical activity as a way to reduce the complications of the disease [7, 8] are very recent. It was believed that physical efforts, by triggering metabolic changes, increasing the body’s oxygen demand and serum lactate, and favoring dehydration, would have the potential to accentuate tissue hypoxia and precipitate vasocclusive crises. In addition, pathological conditions such as anemia, cardiac remodeling and pulmonary hypertension are highly prevalent in this population and further increase the fear of prescribing physical exercises [9]. Most publications that investigated the effects of physical exercise on hemoglobin S patients were based on a short-term exercise program [10]. An innovative, prospective, randomized, multicenter study [11] demonstrated that eight weeks of chronic resistance training exercises was safe for a small group of sickle cell patients, without serious complications during this period. The training group patients showed improvement in physical capacities and muscle metabolism. In addition, they presented a 17% increase in muscle capillary density and increased activity of some oxidative enzymes. Although these works are a breakthrough, there is great difficulty in implementing supervised and properly monitored exercise programs, due to the fact that most patients with sickle cell disease live in developing countries. Therefore, alternative strategies, as home-based exercise program, should be investigated for application on a larger scale.

In contrast to center-based, a home-based exercise program could be an option for patients to perform activities in order to maintain or increase therapeutic effects. A home-based exercise program relies on remote coaching with indirect exercise supervision, mostly or entirely outside of the traditional center-based setting [12]. Home exercises should be designed for safety, practicality, accessibility, and feasibility. Moreover, the patient should perform them without real-time supervision. Home-based exercise programs could be equally effective as supervised center-based cardiac rehabilitation, improving clinical outcome in patients with a low-risk for further events after myocardial infarction on revascularization [13].

Most of our patients live in cities relatively distant from our center, which makes transport difficult for daily or frequent based-center activities. Moreover, high financial costs mean that access to a fitness center is unviable for them. Based on previous reports showing the benefits and safety of physical exercises in sickle cell patients, we hypothesized that a home-based program could promote similar benefits safely in these patients.

## Objective

This study aimed to evaluate the cardiovascular function parameters from an eight-week home-based regular exercise program in patients with sickle cell disease.

## Methods

### Study design

We performed a prospective, non-randomized clinical trial involving patients with SCD from a unique university hemoglobinopathy outpatient medical center located in a county city in Sao Paulo, Brazil. All patients who had periodic consultations at the Service were invited to participate in the Project, taking advantage of the moment when they waited for the routine consultation at the Medical Service. The invitation to participate in the study was carried out by the service physician together with the Physical Education Teacher responsible for guiding and monitoring the execution of the Exercise Protocol. The protocol for this study was approved by the Ethics Committee of the Botucatu Medical School, São Paulo State University (UNESP) under protocol number 20612913.5.0000.5411 (Plataforma Brasil) on February 6, 2014, and registered with the Brazilian Registry of Clinical Trials (RBR-29X8QK). There were a delay in registering this study after enrolment of participants started due to mistakes in the moment of electronic platform submission and difficulties in the resolution. The authors confirm that all ongoing and related trials for this intervention are registered.

The inclusion criteria were: patients older than 18 years, free from sickle cell crises for at least previous 30 days, and agreement to sign an informed consent form. The exclusion criteria were don´t be under treatment for infection at the time of inclusion, diagnosis of moderate or severe pulmonary hypertension, pregnancy, and biomechanical limitations. All participants signed the written consent form after receiving the information and accepting inclusion in the clinical trial.

Recruitment of participants was between May 15 and July 15, 2014. Follow-up was between 16 July and 30 April 2016. From the voluntary expression of interest in participating or not in the Exercise Program, according a quasi randomized trial strategy, the volunteers were allocated into two groups:

Exercise group (EXE): prescription of a regular home-based aerobic exercise program, one hour per day, at least three times, not exceeding five times per week, for eight weeks [14, 15]. The worksheets with the exercise protocol were delivered together with monitoring report forms utilized to log activity performance. Follow-up was performed weekly by phone and at outpatient appointments, when the patients delivered the completed report forms. In the exercise protocol, the participants performed a warm-up, followed by 10 minutes of calisthenics, flexibility, and aerobic exercises. During the first two weeks, they performed 35 minutes of walking between 60% and 70% of the maximum heart rate (HRmax). The HRmax value was determined by the treadmill test. In the third and fourth weeks, 40 minutes of walking between 60% and 70% of HRmax; in the fifth and sixth weeks, 40 minutes of walking between 65% and 75% of HRmax; in the seventh and eighth weeks, 50 minutes of walking between 65% and 75% of HRmax. At the completion of the exercise session, calisthenics and flexibility exercises were performed for 10 to 15 minutes.

Control group (CON): no prescription of physical exercise but instructed to maintain their routine daily activities for a period of 8 weeks.

Patients in both groups underwent clinical evaluation, treadmill test, and transthoracic echocardiogram before (M0) and after (M2) the 8-week protocol. Instructions and the spreadsheet on regular aerobic exercises were provided to the EXE group participants.

All used methods were considered with good validity and reliability for outcome measures.

### Clinical evaluation

The clinical evaluation was carried out by a hematologist, including a physical examination, review of medications, and the collection of demographic and laboratory characteristics.

### Treadmill test

We used the modified Bruce protocol test to evaluate patients’ functional capacity, which was done by a cardiologist blinded to group assignment using an Inbramed® treadmill and Apex 1000 TEB® system with 12 classic leads and CM5 derivation. The test was interrupted at maximum effort or at the development of limiting symptoms by the individual. Modified Bruce protocol is validated to assess functional capacity and to investigate ischemia in sedentary and sick individuals [16–18]. The variables for functional capacity assessment were:

- Duration of the test
- Distance traveled on the treadmill test
- Metabolic equivalent (MET) on the treadmill test (20).

The metabolic equivalent (MET), multiple of the baseline metabolic rate, is equivalent to the energy needed by an individual to remain at rest, represented in the literature by the oxygen consumption (VO2) of approximately 3.5 ml/kg/min. When the energy expenditure is expressed in METs, the number of times by which the rest metabolism was multiplied during an activity is represented. For example, cycling at four METs implies in caloric expenditure four times higher than what it is at rest.

Other variables evaluated were: heart rate, systolic and diastolic blood pressure, and ST segment depression, considering the Y point). There are few studies using treadmill test in sickle cell patients [7, 19]. The entire evaluation protocol followed the recommendations of the American Thoracic Society—2003 [14] and American College of Sports Medicine– 2011 [15]. The treadmill test was considered safety and with good reproducibility in these patients.

### Transthoracic echocardiogram

The ultrasound equipment used was a GE Vivid 6S, with phased-array transducers ranging from 2.5 to 3.5 MHz. The standardizations and techniques recommended by the American Society of Echocardiography were followed [20]. This method is also recommended and validated to evaluate patients with sickle cell disease, with good feasibility and reliability [21–25]. The following variables were obtained: left ventricular diastolic (LVDD) and systolic (LVSD) diameters, left atrium diameter, E and A waves of mitral flow, E’ waves of mitral tissue Doppler imaging (medium of lateral and septal mitral annulus), indexed left atrium volume obtained by the Simpson method, ejection fraction by the Simpson method, S’ wave of mitral tissue Doppler, and estimated arterial pulmonary pressure by tricuspid regurgitation.

### Statistical analysis

Continuous variables are reported as means and standard deviations or medians and interquartile ranges. Categorical variables are reported as proportions. The comparison of categorical data between groups were performed using chi-square tests. The comparisons between groups were performed using two-way ANOVA, considering the effect of time (within-subjects comparison) and group (between subjects comparison) and the interaction between them (time * group). Correlations between the clinical and morphological variables of the same group were evaluated by Pearson test for variables with a normal distribution or Spearman’s test for variables with a non-normal distribution. In all cases, the level of significance was p<0.05. The analysis were conducted using IBM SPSS statistics program.

The program G*Power 3.1.9.2 was utilized to perform the sample size calculation. An improvement of 10% in the LV ejection fraction and the reduction of 10% of E’ Mitral wave and covered length values were defined as the positive response variables. These variations were based on a pre-study statistical power calculation with α (type I) error level set at 0.05. The analysis showed the need for at least 22 patients, 11 per group, to reach sufficient statistical power (1-β err prob) of 0.80. The analysis followed intention to treat principle.

## Results

**Fig 1:**
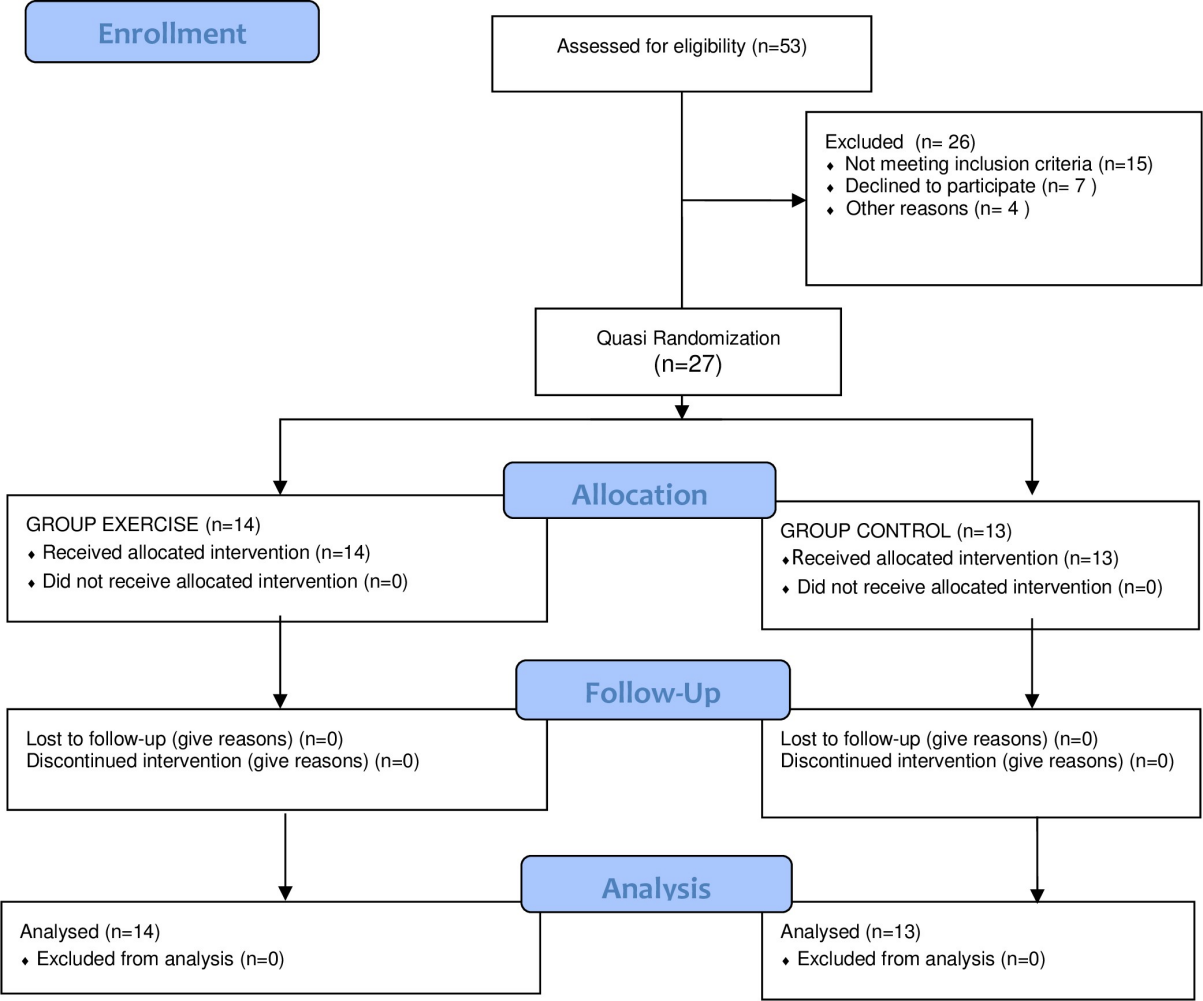
CONSORT 2010 flow diagram.

Fifty-three individuals with SCD were considered for inclusion; of them, 17 did not meet the inclusion criteria, seven could not participate in the study due to locomotion and labor-associated barriers, two died before starting the exercise protocol, and two required hospital admission in the pre-evaluation period. Therefore, we included 27 patients, allocated into the two study groups as: EXE (n = 14) and CON (n = 13). The flow diagram (according to CONSORT 2010) is shown in Fig 1.

The clinical (sickle cell entities, blood transfusion requirements, previous hospitalizations), demographic and laboratory characteristics of both groups are described in Table 1.

**Table 1:** Comparison of the demographic, clinical and laboratory characteristics of the control (CON) and exercise (EXE) groups at the initial moment of the study (M0).

Variable	CON (n = 13)	EXE (n = 14)	p
Age (years)	29.8 ± 10.2	29.1 ± 8.6	0.57
Gender (Men/Women) (n/%)	8 (61.5%)/ 5 (39.5%)	5 (35.7%)/ 9 (64.3%)	0.78
Race (Afro descendant) (n/%)	13 (100%)	14 (100%)	-
Smoking	-	-	-
Alcoholism	-	-	-
Hemoglobin (g/dl)	9.3 ± 1.9	9.6 ± 2.2	0.81
Drugs			
• Hydroxyurea (n/%)	3 (23.1%)	5 (35.7%)	0.17
• Folic Acid (n/%)	13 (100%)	12 (87.5%)	0.16
BMI (Kg/m^2^)	22.2 ± 5	20 ± 4.3	0.55
Entities			
• SS (n/%)	8 (61.5%)	7 (50%)	
• SC (n/%)	3 (23.1%)	3 (21.4%)	0.22
• Sβ0 (n/%)	1 (7.7%)	4 (28.6%)	
• Sβ+ (n/%)	1 (7.7%)	-	
Transfusions			
• Not transfused (n/%)	5 (38.5%)	5 (35.7%)	0.36
• 1 to 10 transfusions (n/%)	3 (23.1%)	7 (50%)	
• + than 10 transfusions (n/%)	5 (38.5%)	2 (14.3%)	
Hospitalizations			
• Without Hospitalizations (n/%)	1 (7.7%)	6 (42.8%)	0.17
• 1 to 10 Hospitalizations (n/%)	8 (61.5%)	2 (14.4%)	
• + than 10 Hospitalizations (n/%)	4 (30.8%)	6 (42.8%)	

Values presented in mean ± standard deviation or number of individuals and percentage. BMI = Body Mass Index; SS (Sickle Cell Homozigous Anemia); SC (Hemoglobinopathy SC); Sβ0 (Sickle Cell Beta 0 Thalassemia Double Heterozigous); Sβ+ (Sickle Cell Beta 0 Thalassemia Double Heterozigous) SAH = Systemic Arterial Hypertension; DMII = Type 2 diabetes mellitus; CAD = Known Coronary Artery Disease. Continuous variables were compared by Student T Test. Significance level P <0.05.

At baseline (M0), no difference was observed between groups CON and EXE regarding to age, gender, Hemoglobin level, Hydroxyurea and Folic Acid Use, Body Mass Index, Sickle Cell Entities, Transfusion and Previous Hospitalization. All the patients were afro-brazilian people. No patient had tobacco use or alcoholism.

For the outcome variables, values before M0 and after M2, the intervention period was compared. The results of the treadmill test are presented in Table 2.

**Table 2:** Comparison of the variables of the ergometric test between the groups (CON and EXE) at time points M0 and M2.

Variable	CON n = 13		EXE n = 14		P
	M0	M2	M0	M2	Interaction Time*Group
Resting HR (bpm)	76±3	76±3	80±3	72±3	0.23
Maximum HR (bpm)	163±4	166±3	179±3	182±1.4	0.95
Resting SBP (mmHg)	109±3	114±3	115±2	114±3	0.32
Maximum SBP (mmHg)	155±5	157±5	161±4	165±4	0.84
Distance (m)	552±160	586±217	809±232	1020±253	<0.01
% of patients with ST-segment depression (n)	46.2±5 (6)	46.2±5.1 (6)	35.7±9 (5)	14.3±3.6 (3)	0.07

Values presented as mean ± standard deviation. HR = heart rate; SBP: systolic blood pressure; distance: maximum distance traveled during the test; ST-segment depression = % of patients with; ejection fraction: indicative of myocardial ischemia. Comparisons were performed using two-way ANOVA with p <0.05.

The mean values for distances walked differed statistically considering the interaction time and group. (p<0.01).

No significant differences in the morphological variables were observed by transthoracic echocardiogram (Table 3).

**Table 3:** Comparison of functional echocardiographic variables between the groups (CON and EXE) at time points M0 and M2.

Variable	CON		EXE		p
	n = 13		n = 14		
	M0	M2	M0	M2	Interaction Time*Group
***LV Systolic Function***					
Tissue Mitral S	9±2.3	13.1±5	9.2±1	9.8±2	0.04
Ejection Fraction %	60.9±10	63.0±9	66.8±7	74.0±6	<0.01
***LV Diastolic Function***					
Mitral E (cm/s)	94.1±24	92.5±25	91.8±22	96.1±25	0.40
Mitral A (cm/s)	53.2±14^a^	57.5±15	50.9±18	53.7±15	0.69
E/A	1.82±0.5^a^	1.64±0.2	1.92±0.6	1.88±0.6	0.42
Mitral E’ (cm/s)	11.9±3	11±2	11.6±3	13.6±2	0.04
Mitral E/E’	8.2±1.4	8.5±2	8.5±0.6	7.1±2	0.38
LAV (mm/m^2^)	32.4±10	32.5±10	27.9±8	31.9±12	0.04
LA Diameter (mm)	36.8±1.4	35.1±1.4	36.3±1.4	37.5±1.4	0.31
***RV Systolic Function***					
Tricúspid S (cm/s)	14.5±3	13.2±3	14.1±3	15.7±3	0.07
Strain	-18±0.7	-18±1.1	-18±1.5	-17±0.9	0.78
***RV Diastolic Function***					
Tricuspid E (cm/s)	53.5±18	61.1±20	56.4±13	56.3±15	0.18
Tricuspid A (cm/s)	38±9	40.4±10	38.4±8	35.4±9	0.93
Tricuspid E’ (cm/s)	14.6±3.4	12.6±2.6	13.8±3.3	15.0±3.4	0.06

Values presented as mean ± standard deviation. Tissue Mitral S: displacement velocity of the mitral annulus at systole (cm/s); FSVE; Mitral E: filling velocity in the mitral Doppler in the fast filling stage; Mitral A: filling velocity at mitral Doppler at atrial contraction; LAV = left atrium volume calculated by the Simpson method indexed to the body surface; LA: left atrium, RV = right ventricle; Tricuspid S = velocity of tricuspid ring displacement in the period of systole of RV; Tricuspid E and A = tricuspid Doppler filling velocity in the fast filling and atrial contraction phases, respectively; Tricuspid E’ = velocity of tricuspid ring displacement in the fast filling phase. Analysis performed using two-way ANOVA with P <0.05.

Regarding the functional parameters, there was an interaction between time and group in relation to increased ejection fraction in the EXE group (p<0.01). The left ventricular diastolic function variables presented an interaction between time and group as to reduced left atrium volume index and increased E’ mitral tissue Doppler in the EXE group (p<0.05).

Notified complications during the performance of the cardiovascular tests was a painful episode occurred in one patient of the CON group after the treadmill test at M0, followed by improvement within a few hours after treatment with analgesics and oral hydration. In the two Groups, there was no related infectious episode or painful crisis or hospitalization during the eight weeks evaluation period.

## Discussion

In the present study, we present a home-based exercise protocol applied to patients with Sickle Cell Disease of a Brazilian Hemoglobinopathy Center, prescribed according to the recommendations of the American Thoracic Society—2003 [14] and the American College of Sports Medicine—2009 [15]. These guidelines recommend for the general population aerobic activities for at least 120 minutes per week, divided between 3 to 7 days per week. Based on these references, we developed an exercise protocol according the functional adaptations of the individuals over the training course. In our cohort, the choice of a home-based exercise program was due to the difficulty of supervising patients on the spot at least right times a week, and the fact that most of them live in distant cities (20 to 100 km away). This type of program may be the easiest to implement on account of the low cost of its services and avoidance of patient travel. During the prescribed home-based exercise period, the EXE group underwent aerobic training with progressive load increase. Intensities were individually prescribed.

Our hemoglobinopathy center is a regional service include in the Brazilian Unified Health System, serving about 30 cities, and it is the only public reference service in our region for SCD patients. In our study, the limited patient population precluded the grouping of patients by entities or potential modulators of disease severity. In addition, randomization was not possible, so the groups were formed based on the voluntary disposition of patients to practice home-based exercises. On the other hand, the sample was composed of a single service unit where patients were followed by a multiprofessional team.

About the exercise protocol, the range between 60% and 70% of the maximum heart rate (HRmax) determined by the treadmill test for the first four weeks was chosen to provide feasibility and avoid ischemia induction. The patients were young in the average and in according to treadmill test results, they tolerated well a longer distance in the test. They could walk 30-50min with safety and tolerability. All applied treadmill and echocardiographic protocols were based on studies that evaluated patients with sickle cell disease in order to ensure safety and feasibility [7, 19, 22–25].

Comparison between baseline variables (M0) showed the EXE and CON groups paired by age, sex and race presented no significant difference in demographic or clinical variables, Hemoglobin, use of hydroxyurea, BMI, sickle cell disease entities, previous transfusion, or hospitalization (Table 1). The patient’s decision to participate in the EXE group, therefore, was a personal choice, and was not determined by the severity of the disease, pathological entity, frequency of infections, previous hospitalizations and transfusions, or use or not of hydroxyurea. None of the patients in the EXE Group reported the onset of events or complications of the disease during the weeks of practice of the Exercise Program. These results suggest the present home-based exercise program is safe and possible to be applied to patients with sickle cell disease, regardless of the number of previous transfusions or hospitalizations or the patient’s pathological entity.

Despite the short term duration (8 weeks), our findings suggest that the home-based physical training effectively improved cardiopulmonary function and tolerance of effort. The variables related to the treadmill test (Table 2) showed an improvement in functional capacity in the EXE group, verified by the increase in the distance covered (p<0.01).

Regarding the echocardiographic findings (Table 3), we observed an improvement in systolic function, evaluated by ejection fraction and diastolic function expressed by the indexed left atrium volume and E’ wave of mitral tissue Doppler.

We found a relatively elevated frequency of ST depression in the treadmill test at M0, probably secondary to endothelial dysfunction. Although it did not reach statistical significance, the normalization of the ST segment in 3 of the 5 patients with previous ST depression in the EXE group suggests an improvement in myocardial perfusion after carrying out the exercise program.

Recent studies corroborated the benefits of exercise in healthy individuals [26] and in patients with diseases other than SCD [27]. Favorable effects on cardiovascular function are associated with improvements in oxidative stress, inflammatory processes, and blood rheology. Regular physical training for a sufficient period may promote greater vasodilatory activity [28], increasing endothelial nitric oxide synthase activity, and, in turn, endothelial nitric oxide bioavailability, suggesting a reduction in oxidative stress concomitant with peripheral vasodilation, resulting in an improvement in compliance and cardiac output resulting in better exercise tolerance. Also, regular physical exercise decreases the chronic inflammatory process by the endothelial modulation of certain adhesion proteins, such as E-selectin, V-CAM, and I-CAM [29, 30], in addition to decreasing leukocyte infiltration, as observed in sickle cell experimental studies [31, 32]. It is interesting to observe the complementarity between the benefits that exercise offers and the changes related to the sickle cell disease vasculopathy [33], that is, abnormal adhesion of the vascular endothelium, the pro-inflammatory state, the abnormal adhesion of leukocytes and platelets, consumption of nitric oxide, increase in vasomotor tone and the luminal narrowing secondary to the proliferation of smooth muscle cells. In this sense, it is clear that physical exercise is potentially an excellent adjuvant treatment for patients with sickle cell disease [34].

Despite therapeutic advances in recent years, SCD patients still experience significant morbidity and mortality [35, 36]. Poor socioeconomic conditions, inactivity, and a sedentary lifestyle are frequently observed in those patients, with a potential negative impact on quality of life and occupational well-being [37].

Until recently, the practice of regular physical exercise by SCD patients was not encouraged because of the possibility of triggering painful attacks or exacerbating anemia symptoms [38]. The recommendation for patients with SCD was the practice of light activities, with slow and progressive load increase [6].

Feasibility and safety have been demonstrated in a 12-week home aerobic exercise program Clinical Trial in children with Sickle Cell Anemia [39]. The exercises were performed using a home stationary bicycle. Because of reduced adherence to training, Peak VO2 and other exercise parameters did not significantly improve at the end of the study.

Safety in performing moderate endurance exercise in adult patients was demonstrated in a multicenter randomized study, which demonstrated that the exercise program provided improved functional capacity, muscle capillary density, and oxidative enzyme activity in sickle cell patients without severe chronic complications [11, 40]. The same study group demonstrated through the blood lactate curve based monitoring that submaximal incremental exercise strategy constituted a safe procedure [41]. Another Adult Sickle Cell Anemia Patients Clinical Trial applied a 12 weeks protocol. Their study demonstrated a reduction in TNF-α (tumor necrosis factor–alpha_ and IL-6 (Interleukin-6) in the Exercise Group [42]; the training group preformed aerobic exercise training on the treadmill and all training sections were supervised.

Regarding Clinical Trials for Sickle Cell Patients, no prospective study has evaluated the cardiovascular response in a regular exercise program longer than three days in this population [6].

Despite limitations of the our study design, particularly regarding group selection, sample size, and quasi-randomization, our study assessed a population of patients treated at a single service unit by a single professional team. This home-based program proved to be applicable in the daily routines of patients with SCD. The eight-week duration of the home-based exercise protocol was able to demonstrate improvement in functional capacity and cardiovascular function, without triggering painful crises or worsening of SCD symptoms. We believe that the main clinical implication of our study results is the evidence that exercise (and its widely known benefits), with adequate prescription and monitoring, should be extended to the patients with sickle cell disease who wish start it.

## Conclusion

This study demonstrated a favorable impact on treadmill test and echocardiographic parameters after the eight-week exercise training program for patients with SCD using a home-based program. Further studies will be needed to establish the best and safe exercise practices for the benefit of patients with sickle cell disease.

## Supporting information

S1 Checklist (PDF)

S1 File (DOCX)

S2 File (DOC)

## List of abbreviations

SCD: Sickle cell disease
FC: Functional capacity
EXE: Exercise group
CON: Control group
M0: Before the 8-week protocol
M2: After the 8-week protocol
HRmax: Maximum heart rate
LVDD: Left ventricular diastolic diameter
LVSD: Left ventricular systolic diameter
