# Supplementary material for: Cardiovascular benefits of a home-based exercise program in patients with sickle cell disease
Source: PLoS One. 2021 May 12;16(5):e0250128. doi: 10.1371/journal.pone.0250128 (PMC8115779; doi:10.1371/journal.pone.0250128)
Supplement: S1 File — (DOCX) [file pone.0250128.s002.docx]

**Inclusion criteria:**

• Patients with a diagnosis of sickle cell anemia (standard SS or SC) who agree to participate in the study, and agreeing and signing informed consent form (IC) -

• Age over 18 years, of both sexes

**Exclusion criteria:**

• Individuals who experienced a painful crisis in the last 30 days, at the time of inclusion

• patients with recurrent infections or daily painful crises that limit physical exercise.

**STUDY PROTOCOL**

After inclusion, patients will choose to participate in one of the two groups (self-selection), the exercise group (EXE) and the control group (CON). Initially, we had thought about doing a randomization of the groups. Whereby the intervention requires adherence to the exercise program, we modified it for the self-selection of the groups. The EXE group will receive a prescription for regular physical exercise, instructed to perform them 3 times a week, at least one of them supervised by the physical educator, cardiologist and physiotherapist, for a period of 4 weeks. The CON group will not receive specific guidance for exercising, continuing its routine activities and standard outpatient follow-up.

Initially, all research subjects will undergo clinical evaluation (Annex I), physical evaluation and will answer the standard SF-36 questionnaire to define quality of life. The transthoracic echocardiogram, exercise test, carotid ultrasound and the 24-hour holter will also be performed in a period not exceeding 48 hours. During this same period, blood samples will be taken to measure metabolic and inflammatory markers. All procedures are described in detail below.

These same assessments will be repeated at two and four months, according to the scheme illustrated in Figure 1.

**Design**

**Final of**

**Physical activity**

***Start of physical*** ***activity***

***Evaluation***

***2 months***

**f**

***4 months***

***4***

**f**

***Evaluation***

***Screening and Evaluation***

**EXE**

**EXE/CON**

**EXE/CON**

**Group Inclusion**

**EXE/CON**

***Exercise Program***

**Figure 1:** Experimental Design

**Clinical and Physical Evaluation**

The clinical evaluation must be performed by the hematologist, and will consist of a clinical record and general and special physical examination, medications, treatments performed.

The anthropometric evaluation will consist of measurements of body weight (BW) and height (E) to determine the Body Mass Index (BMI), in addition to the abdominal circumference. To measure body weight, a platform scale (Filizola) with a maximum capacity of 150 kilos and 0.1 kg accuracy will be used. At the time of weighing, patients should be in light clothing and without shoes. To measure height, a portable stadiometer with a precision of 0.1 cm will be used, considering the final result as the arithmetic mean of 3 consecutive measurements. The PC and E values ​​will be used to calculate the BMI to diagnose the nutritional status of individuals. The BMI value is extracted from the formula: BMI = PC / E2 and the classification of the nutritional status given according to the guidelines of SISVAN (2008) .14 The diagnosis of overweight will be established when the BMI presents values ​​between 25 to 30 Kg / m2 and for the diagnosis of obesity the BMI values ​​must be equal to or greater than 30 (SISVAN 2008). Waist circumference will be measured between the midpoint of the last rib and the iliac crest (McCarthy, 2001) using a non-extensible measuring tape, with the patient in a supine position, after complete expiration.

The adipose mass and lean mass will be assessed by electrical bioimpedance using a portable device model BIA 101Q with electric current of 800 microA and 50 kHz with Bioimpedance Body Assessment Program (serial number - 93236, version 2.5, brand QUANTUM RJL SYSTEMS) . The analysis will be carried out on the dominant side of the individual with the same fasting overnight for 8 to 12 hours.

**Assessment of motricity capacity**

Regarding the assessment of motor skills, the following tests will be carried out: wingspan and flexibility. These tests will be carried out according to the standardization proposed by Projeto Esporte Brasil (PROESP-BR 2007). To determine cardiovascular resistance and the intensity of aerobic exercises, an exercise electrocardiogram will be performed.

The wingspan will be measured by a measuring tape fixed to the wall parallel to the floor. Patients will position themselves facing the wall, with their arms abducted at 90 degrees in relation to the trunk. The elbows should be extended and the forearms supinated. The individual should position the end of the left middle finger at the zero point of the measuring tape, measuring the distance to the end of the right middle finger.

To determine joint amplitude, the sit and reach test on the Well bench will be used. Barefoot individuals will sit facing Wells' bench with their legs extended and joined. They will place one hand on the other and raise their arms vertically. Leaning the body forward until reaching the fingertips as far as possible, on the graduated ruler, without flexing the knees and without using rocking movements. Each patient will make two attempts.

**Treadmill test**

The research subjects will be submitted to a maximum standard exercise test, performed by a single experienced examiner, using an Inbramed® treadmill and Apex 1000 TEB® system with 12 classic leads and CM5. The modified Bruce protocol will be used, with the test interrupted at the limit of physical capacity reported by the individual or in the presence of symptoms that require the examination to be interrupted. The post-exercise recovery phase was 6 minutes. Systolic and diastolic blood pressure will be measured by means of a mercury column sphygmomanometer and appropriate cuff, brand Takaoka®, with the patient lying down, before the beginning of the effort and during recovery, and in an orthostatic position, during the last minute of each effort stage

**Echocardiogram**

A complete echocardiographic study will be performed in all patients considered before and after the combined exercise program in the intervention group and in the same period listed in the control group. The ultrasound equipment used will be GE Vivid 6S, with phased-array transducers, with frequencies ranging from 2.5 to 3.5 MHz. In the echocardiographic study, the standards and techniques recommended by the American Society of Echocardiography25 will be considered. Monodimensional images obtained with the ultrasound beam guided by the two-dimensional image will be analyzed, obtained with the transducer in the parasternal position of the major axis. The image of the left ventricular cavity will be obtained by positioning the M-mode cursor just below the mitral valve plane between the papillary muscles. The images of the aorta and the left atrium will also be obtained in the parasternal position of the major axis with the M-mode cursor passing through the valves of the aortic valve. The measurements, in centimeters, of the diastolic diameter of the left ventricle (LVDD), systolic diameter of the left ventricle (LVDD), thickness of the posterior wall (PP), diameter of the left atrium (LA) and diameter of the aorta (AO) will be performed, through the cursor of the equipment itself, during the examination. Three to five consecutive cycles will be used to perform the measurements, obtaining the arithmetic mean afterwards. The images of the ventricular chambers' records, mode M, will be archived for eventual later consultations. The diameter of the left ventricular outflow tract (LVS, cm) and the ascending aorta will be obtained in the parasternal window, in two-dimensional mode.

The transmittal diastolic and systolic transvalvular aortic flows will be obtained with the transducer placed in the apical positions four and five chambers, respectively, allowing the measurements of wave E (E, cm / s), wave A (A, cm / s), maximum speed blood flow in the left ventricular outflow tract (AVO, cm / s) and the integral time-velocity (VTI) in the left ventricular outflow tract. Heart rate (HR) will be estimated by the time between two consecutive beats. From the Doppler visualization of the mitral and aortic valves, the following will be calculated: isovolumetric relaxation time (IVRT), defined as the time interval between the end of the aortic valve flow and the beginning of the mitral transvalvular flow and the E wave deceleration time. (TDE).

The Tissue Doppler (TDI) image will be obtained in real time, in the apical window of four chambers. The volume sample will be placed in the basal portion of the ventricular wall (mitral annulus), interventricular septum and basal portion of the right ventricular wall (tricuspid annulus). The angle of incidence between the ultrasound beam and the ventricular wall or septum must be less than 30º. Peak annular velocities will be measured in early diastole (E´), atrial contraction (A´) and in systole (s´).

The measurements related to the flows will also be performed directly on the echocardiograph monitor, following the same system described above.

The volumes of the left and right atria will be obtained from the planimetry in the 4-chamber apical window.

The LV ejection fraction will be calculated using the Simpson method.

The other variables derived from mathematical calculations, based on the measurements obtained, are described below:

• AE / AO

•% E = [(DDVE - DSVE) / DDVE] x 100 (%), where% E is the percentage of left ventricular shortening

• AND THE

• AAE / AAD

• SIV + PP / DDVE = left ventricular relative thickness

•% E = [(DDVE-DSVE) / DDVE] x100 (%), where% E is the percentage of left ventricular shortening

• AND THE

• AND IS

• DC = (VSVE) 2 * 0.785 x VTI x HR, with DC being the cardiac output (volume of blood flow through the aortic valve per minute;

• LV mass indexed to the body surface: 0.8 x {1.04 [(DDVE + SIV + PP) 3 - (DDVE) 3]} + 0.6 g / body surface

• PAP = maximum gradient of the tricuspid transvalvular reflux when present + estimated pressure in the right atrium, with PAP = estimated systolic pressure in the pulmonary artery.

• Deformation (strain,%)

• Strain rate (strain rate, s-1)

**Carotid Ultrasound**

Carotid ultrasound examinations will be performed by an examiner, using the General Electric (GE) Vivid S6 equipment, equipped with a 7.0 MHz linear ultrasonic transducer and an image recording system. Patients will remain in the supine position with their head slightly tilted to the side contralateral to the studied carotid. The intimal-medium thickening will be obtained by the automated method, with determination of the maximum and average thickness, using Software developed by GE. The measurements will be carried out on the posterior wall of the common carotids, left and right; the average between the two measures will be considered. The images will be obtained and analyzed following the recommendations of the “Consensus Statement from the American Society of Echocardiography” 26 and the “Mannhein Carotid Intima-Media Thickness Concensus (2004-2006) 27.

Atherosclerosis plaque research will also be carried out, and when they are present, they will be classified according to echogenicity, using the criteria proposed by Gray-Weale28

• Type I: hypoechoic plaques;

• Type II: predominantly hypoecoidal plaques;

• Type III: predominantly hyperechoic plaques;

• Type IV: hyperechoic plaques;

• Type V: calcified plate with acoustic shade.

The more hypoechoic (type I) the plaques are, the greater their relationship with cardiovascular events.

**Quality of life assessment**

Patients will be asked to complete a questionnaire on health-related quality of life, the Medical Outcomes Study 36-item Short-Form Health Survey (ANNEX 3), to verify the impact of the PE program on this aspect29.

**Evaluation of metabolic biomarkers**

Blood samples should be collected with anticoagulants obtained after a 12-hour overnight fast, by means of venipuncture in a closed vacuum system Vacutainer® (Becton Dickinson) 30,31.

• Plasma glucose: Glucose will be quantified using Johnson's commercial kit - USA.

• Triacylglycerol: Triacylglycerol will be analyzed by the colorimetric enzymatic method

• Total cholesterol: Total cholesterol will be analyzed by the calorimetric enzymatic method.

• HDL-Cholesterol: HDL-cholesterol will be quantified by a precipitation method (Lopes-Virella et al, 1977).

• LDL-Cholesterol: The calculation of LDL-cholesterol will be performed using the Friedewald formula for triacylglycerol below 400.0 mg / dL. (NCEP - ATP III 2001) 31.

• LDL-cholesterol = Total cholesterol - HDL-cholesterol - triglycerides

5

• Uric Acid: Uric acid will be quantified by the calorimetric enzymatic method

• CBC: The CBC will be performed by an automated system using flow cytometry.

**Evaluation of inflammatory biomarkers and BNP**

The blood collection, for the measurement of BNP in plasma, will be done by venipuncture, where a 10 mL sample of venous blood will be collected directly in a tube of the Vacutainer® system (Becton Dickinson), containing heparin and properly identified. The blood sample will be immediately centrifuged for 10 minutes at 2000 rpm and at room temperature, to obtain plasma, the volume of which will be aliquoted and stored at -80ºC until the moment of determining the concentrations of biomarkers.

The determination of BNP in plasma will be performed using a competitive enzyme immunoassay (ELISA), carried out according to the manufacturer's instructions (Wuhan EIAab, Science Co., Ltd, China, cat. E0541h). Initially 50 μL of the standard BNP solution or plasma sample will be added to the holes in the plate. Detection solution A (50 uL) will be immediately added to the holes and the plate incubated for 60 min at 37oC. Then the plate will be washed four times with buffer solution and then the reaction will be incubated with 100 μL of the developing reagent for 45 min at 37oC. After a new wash cycle of the plate, 90 µL of the substrate solution will be added to each orifice and the plate will be incubated at room temperature and protected from light for 20 min. The reaction will be blocked by the addition of 50μL of 2M sulfuric acid and the plate will be read in an ELISA reader (Multiskan EFLAB, Helsinki, Finland) with a wavelength of 450 nm. The sensitivity limit of the kit will be 3.90 pg / mL.

**Determination of TNF-α, IL-1 β, IL-6, IL-10 and C-reactive protein cytokines**

Quantikine ELISA commercial kits will be used to quantify cytokines and C-reactive protein (R&D Systems, Minneapolis, MN, USA). The reactions will be developed according to the manufacturer's instructions and described according to the technique below.

96-hole, flat-bottomed plates (MaxiSorp-Nunc Life Tech. Inc., Maryland, MA, USA) previously sensitized with specific anti-cytokine or anti-PCR monoclonal antibody will receive 200μL of plasma or recombinant cytokines (R&D Systems ). After 2 hours of incubation at room temperature the plate will be washed four times with buffer solution and then incubated with 200 μl of polyclonal anti-cytokine or anti-PCR developer antibody (R&D Systems), followed by incubation for 2 hours at room temperature . The plate will be washed again and 100μL of streptoavidine conjugated to peroxidase (R&D Systems), at a concentration of 2μg / mL for 20 min at 37ºC, followed by washing the plate with PBST. After this period, 100μL of the enzymatic substrate will be added, consisting of stabilizing solutions of hydrogen peroxide and tetramethylbenzidine (DY999 - R&D Systems). The plates will be incubated at room temperature, protected from light, for 20 minutes and the reaction will be blocked by the addition of 50μL of 2M sulfuric acid. The plate will be read in an ELISA reader (Multiskan EFLAB, Helsinki, Finland) with a wavelength of 450nm. The concentrations of cytokines present in monocyte culture supernatants, treated or not with LPS and PGN, will be calculated from the standard curves performed with the different human recombinant cytokines. In the assays, the concentrations of monoclonal and polyclonal antibodies, as well as the specific recombinant cytokines used in the standard curves, will be those recommended by the manufacturer (R&D Systems). The sensitivity limit of the kits will be 1.6 pg / mL for TNF-α, 3.9 pg / mL for IL-1 β and IL-10, 1pg / mL for IL-6 and 0.010 ng / mL for PCR.

**EXERCISE PROTOCOL**

The exercise program will last for 4 months, and during this period individuals will be instructed to practice the activities at least 3 times a week, not exceeding 5 times a week, with at least one of the activities being supervised.

The exercise protocol will be based on low-intensity aerobic activities of increasing duration as individuals improve their fitness.

The exercise protocol will have three moments:

**Initial phase:**

Calisthenic exercises will be performed, suggested during the face-to-face class days (supervised activities), and these exercises will last approximately 10 minutes in order to warm up the muscles to be worked.

**Main part:**

The exercise protocol had initially had an uninterrupted 35-minute walk with an intensity between 60 and 75% of the maximum heart rate determined by the exercise test. With the improvement in physical fitness, the walking time should be gradually increased until reaching 50 minutes of walking.

**Final part**

It will be aimed at stretching and relaxing the muscle groups worked, and these activities will also be oriented on the days of supervised classes.

This exercise protocol recommends low-intensity and long-term activities for individuals who have sickle cell disease, as high-intensity exercises can trigger painful crises and have no evidence of improvement in cardiovascular variables in individuals with coronary artery disease. Another relevant point of this protocol is the importance of stretching and relaxing activities, as it is related to improved quality of life and muscle pain in normal individuals after physical activity.

**STATISTICAL ANALYSIS**

Continuous variables will be presented as means and standard deviations or medians and interquartile ranges. Categorical variables will be presented as proportions. Comparisons between groups will be made using the Student's t-test or Mann-Whitney test or chi-square test. The comparisons between the three moments of the study protocol will be made using ANOVA for repeated measures. The associations between clinical and laboratory variables and the effect of physical exercise prescription will be assessed using linear or logistic regression models or Spearman's Correlation Coefficient. In all cases, the significance level p <0.05 will be adopted.

**Critical analysis of possible risks and benefits**

The research itself does not pose any risk for patients in the control group, since their treatment for possible comorbidities will be carried out in accordance with the consensus already established in the literature and the routine assessments and examinations they will undergo do not offer any additional risk. . No drugs will be added or removed for the purpose of the research. For patients in the intervention group (AFEXE), an imminent risk inherent to the practice of PE is not expected, due to the care in creating the training protocol and constant monitoring of the qualified Physical Education professional (Jonas Alves de Araujo Junior) in the examination sessions. physicist and cardiologist and hematologist (Meliza Goi Roscani and Newton Key Hokama). Patients will receive adequate hydration and monitoring of vital signs. However, some discomforts, such as increased sweating during PE and acute muscle pain characteristic of the initial training phase, may occur as a result of participating in the PE program. However, in case of symptoms suggestive of joint pain, shortness of breath, limiting tiredness, physical exercise will be stopped immediately. According to the severity of the symptoms presented, the patient will be excluded from the exercise protocol.

**7 – ETHICS**

Patients will be selected and invited to participate in the research, always respecting the free and informed consent term and the rules of the ethics committee. They will be submitted to clinical evaluation and the methodology previously described, after acceptance and signing of the Free and Informed Consent Term (annex 3). The data recorded in the attached clinical record will be analyzed according to the statistical treatment already described and published, preserving the patients' identity and dignity, according to resolutions 196/96 and 251/97 of the National Health Council.

All stable patients, whether in the control group or the intervention group, after the end of the research, if there are any benefits, will be encouraged to regularly practice adequate PE and forwarded, through a report, to networks of gyms or appropriate places for the practice of PE with qualified professionals.
