# Supplementary material for: Cardiovascular benefits of a home-based exercise program in patients with sickle cell disease
Source: PLoS One. 2021 May 12;16(5):e0250128. doi: 10.1371/journal.pone.0250128 (PMC8115779; doi:10.1371/journal.pone.0250128)
Supplement: S2 File — (DOC) [file pone.0250128.s003.doc]

**PROTOCOLO DO ESTUDO**

**Critérios de inclusão:**

- Pacientes com diagnóstico de anemia falciforme (padrão SS ou SC) que aceitem participar do estudo, concordando com o termo de consentimento livre e esclarecido (TCLE)-
- Idade superior a 18 anos, de ambos os sexos

**Critérios de exclusão:**

- Indivíduos que apresentaram crise dolorosa nos últimos 30 dias, no momento da inclusão
- pacientes com infecções de repetição ou crises dolorosas diárias que limitem a realização de exercício físico.

**4 – PROTOCOLO DO ESTUDO**

Após a inclusão, os pacientes escolherão participar de um dos dois grupos, o grupo exercício (AFEXE) e grupo controle (AFCONT). Inicialmente, havíamos pensado em fazer uma randomização dos grupos, mas como a intervenção exige a aderência ao programa de exercícios, modificamos para a formação voluntária dos grupos. O grupo AFEXE receberá prescrição de exercício físico regular, orientados para executá-los 3 vezes por semana, sendo pelo menos uma delas supervisionada pelo educador físico, cardiologista e fisioterapeuta, por período de 4 semanas. O grupo AFCONT não receberá orientação específica para a prática de exercício, continuando suas atividades de rotina e seguimento ambulatorial padrão.

Inicialmente, todos os sujeitos da pesquisa passarão por avaliação clínica (anexo I), avaliação física e responderão ao questionário padrão SF-36 para a definição da qualidade de vida. Também serão realizados em período não superior a 48 horas o ecocardiograma transtorácico, teste ergométrico, ultrassonografia de carótidas e o holter de 24 horas. Nesse mesmo período será colhida amostra de sangue para a dosagem de marcadores metabólicos e inflamatórios. Todos os procedimentos são descritos com detalhes abaixo.

Essas mesmas avaliações serão repetidas aos dois e quatro meses, conforme esquema ilustrado na Figura 1.

**Desenho do estudo**

**Final do**

**atividade física**

***2 meses***

**f**

***4 meses***

***4***

**f**

***Triagem e***

***Avaliação***

**Início da**

**atividade física**

***Avaliação***

***Avaliação***

**AFEXE**

**AFEXE/AFCON**

**Formação dos grupos**

**estudados**

**AFEXE/AFCON**

**AFEXE/AFCON**

***Programa de exercícios***

**Figura 1:** Desenho Experimental

**Avaliação clínica**

A avaliação clínica deverá ser realizada pelo hematologista, e será composta por ficha clínica (anexo I) e exame físico geral e especial, medicações, tratamentos realizados.

**Avaliação física**

A avaliação antropométrica será composta por medidas de peso corporal (PC) e estatura (E) para determinação do Índice de Massa Córporea (IMC), além da circunferência abdominal. Para aferição do peso corporal será utilizada balança tipo plataforma (Filizola) com capacidade máxima de 150 quilos e precisão de 0,1 kg. No momento da pesagem os pacientes deverão estar com roupas leves e sem sapatos. Para aferição da estatura será utilizado estadiômetro portatil com precisão de 0,1 cm considerando-se como resultado final a média aritmética de 3 medidas consecutivas. Os valores de PC e E serão utilizados para calcular o IMC para diagnosticar o estado nutricional dos indivíduos. O valor de IMC é extraído a partir da formula: **IMC= PC/E^2^** e a classificação do estado nutricional dada de acordo com as orientações do SISVAN (2008).^14^ O diagnóstico de sobrepeso será estabelecido quando o IMC apresentar valores entre 25 a 30 Kg/m^2^ e para o diagnóstico de obesidade os valores do IMC deverão ser iguais ou superiores 30 (SISVAN 2008). A circunferência da cintura será aferida entre o ponto médio da ultima costela e a crista ilíaca (McCarthy, 2001) utilizando-se uma fita métrica não extensível, estando o paciente em posição supina, após a expiração completa.

A massa adiposa e a massa magra serão avaliadas por bioimpedância elétrica utilizando um aparelho portátil modelo BIA 101Q com corrente elétrica de 800 microA e 50 kHz com Programa de Avaliação Corporal por Bioimpedância (número de série - 93236, versão 2.5, marca QUANTUM RJL SYSTEMS). A análise será realizada no lado dominante do indivíduo estando o mesmo em jejum noturno de 8 a 12 horas.

**Avaliação da capacidade motora**

Em relação à avaliação da capacidades motoras, serão realizados os seguintes testes: envergadura e flexibilidade. Estes testes serão realizados de acordo com a padronização proposta pelo Projeto Esporte Brasil (PROESP-BR 2007). Para se determinar a resistência cardiovascular e a intensidade dos exercícios aeróbios será realizado um eletrocardiograma de esforço.

A envergadura será aferida por uma trena fixada na parede paralelamente ao chão. Os pacientes irão posicionar-se de frente para a parede, com os braços em abdução em 90 graus em relação ao tronco. Os cotovelos devem estar estendidos e os antebraços supinados. O individuo deverá posicionar a extremidade do dedo médio esquerdo no ponto zero da trena, sendo medida a distância até a extremidade do dedo médio direito.

Para se determinar a amplitude articular será utilizado o teste de sentar e alcançar no banco de Well. Os indivíduos descalços, sentarão de frente para o banco de Wells com as pernas estendidas e unidas. Colocarão uma das mãos sobre a outra e elevarão os braços na vertical. Inclinando o corpo à frente até alcançar a ponta dos dedos o mais longe possível, sobre a régua graduada, sem flexionar os joelhos e sem utilizar movimentos de balanço. Cada paciente realizará duas tentativas.

**Teste ergométrico**

The patients will be submitted to a maximum standard exercise test, performed by a single experienced examiner, using an Inbramed® treadmill and Apex 1000 TEB® system with 12 classic leads and CM5. The modified Bruce protocol will be used, with the test interrupted at the limit of physical capacity reported by the individual or in the presence of symptoms that require the examination to be interrupted. The post-exercise recovery phase was 6 minutes. Systolic and diastolic blood pressure will be measured by means of a mercury column sphygmomanometer and appropriate cuff, brand Takaoka®, with the patient lying down, before the beginning of the effort and during recovery, and in an orthostatic position, during the last minute of each effort stage

**Ecocardiograma**

Estudo ecocardiográfico completo será realizado em todos os pacientes considerados antes e após o programa de exercício físico combinado no grupo intervenção e no mesmo período relacionado no grupo controle. O equipamento de ultrassonografia utilizado será GE Vivid 6S, com transdutores phased-array, com frequência que variando de 2,5 a 3,5 MHz. No estudo ecocardiográfico, serão consideradas as padronizações e as técnicas recomendadas pela American Society of Echocardiography^25^. Serão analisadas imagens monodimensionais obtidas com o feixe de ultra-som orientado pela imagem bidimensional, obtida com o transdutor na posição paraesternal eixo maior. A imagem da cavidade ventricular esquerda será obtida posicionando o cursor do modo-M logo abaixo do plano da valva mitral entre os músculos papilares. As imagens da aorta e do átrio esquerdo também serão obtidas na posição paraesternal eixo maior com o cursor do modo-M passando pelas válvulas da valva aórtica. As medidas, em centímetros, do diâmetro diastólico do ventrículo esquerdo (DDVE), diâmetro sistólico do ventrículo esquerdo (DSVE), espessura da parede posterior (PP), diâmetro do átrio esquerdo (AE) e diâmetro da aorta (AO) serão realizadas, por meio do cursor do próprio equipamento, durante o exame. Três a cinco ciclos consecutivos serão utilizados para a realização das medidas, obtendo-se a média aritmética em seguida. As imagens dos registros das câmaras ventriculares, modo M, serão arquivadas para eventuais consultas posteriores. O diâmetro da via de saída do ventrículo esquerdo (VSVE, cm) e da aorta ascendente serão obtidos na janela paraesternal, no modo bidimensional.

Os fluxos diastólico transmitral e sistólico transvalvar aórtico serão obtidos com o transdutor colocado nas posições apicais quatro e cinco câmaras, respectivamente, permitindo as medidas da onda E (E, cm/s), onda A (A, cm/s), velocidade máxima do fluxo sanguíneo na via de saída do ventrículo esquerdo (VAO, cm/s) e a integral tempo-velocidade (VTI) na via de saída do ventrículo esquerdo. A freqüência cardíaca (FC) será estimada pelo tempo entre dois batimentos consecutivos. A partir da visualização do Doppler das valvas mitral e aórtica, serão calculados: tempo de relaxamento isovolumétrico (TRIV), definido como o intervalo de tempo entre o final do fluxo valvar aórtico e o início do fluxo transvalvar mitral e tempo de desaceleração da onda E (TDE).

A imagem de Doppler Tecidual (TDI) será obtida em tempo real, na janela apical de quatro câmaras. A amostra de volume será colocada na porção basal da parede ventricular (ânulo mitral), septo interventricular e porção basal da parede do ventrículo direito (ânulo tricúspide). O ângulo de incidência entre o feixe de ultrasom e a parede ventricular ou septo deverá ser inferior a 30º. As velocidas de pico anular serão medidas na diástole precoce (E´), contração atrial (A´) e na sístole (s´).

As medidas referentes aos fluxos também serão realizadas diretamente no monitor do ecocardiógrafo, obedecendo a mesma sistemática descrita acima.

Os volumes dos átrios esquerdo e direito serão obtidas a partir da planimetria na janela apical 4 câmaras.

A fração de ejeção do VE será calculada a partir do método de Simpson.

As outras variáveis derivadas de cálculos matemáticos, a partir das medidas obtidas estão descritas abaixo:

- AE/AO
- %E = [(DDVE – DSVE) / DDVE] x 100 (%), sendo %E a porcentagem de encurtamento do ventrículo esquerdo
- E/A
- AAE/AAD
- SIV+PP/DDVE = espessura relativa do ventrículo esquerdo
- %E= [(DDVE-DSVE)/DDVE]x100 (%), sendo %E a porcentagem de encurtamento do ventrículo esquerdo
- E/A
- E/E
- DC= (VSVE)^2^*0,785 x VTI x FC, sendo DC o débito cardíaco (volume de fluxo sanguíneo pela valva aórtica por minuto;
- Massa do VE indexada para a superfície corpórea: 0.8 x{1.04[(DDVE + SIV+PP)3 - (DDVE)3]}+ 0.6 g/superfície corpórea
- PAP= gradiente máximo do refluxo transvalvar tricúspide quando presente + pressão estimada no átrio direito, sendo PAP= pressão sistólica estimada na artéria pulmonar.
- Deformação (strain, %)
- Taxa de deformação (strain rate, s-1)

**Ultrassonografia das Carótidas**

Os exames ultrassonográficos das carótidas serão realizados por um examinador, utilizando-se o equipamento General Eletric (GE) Vivid S6, dotado de transdutor ultrassônico linear de 7,0 MHz e sistema de registro de imagens. Os pacientes permanecerão em decúbito dorsal horizontal com a cabeça ligeiramente inclinada para o lado contra-lateral à carótida estudada. O espessamento médio-intimal será obtido pelo método automatizado, com determinação da espessura máxima e média, utilizando-se Software desenvolvido pela GE. As medidas serão realizadas na parede posterior das carótidas comuns, esquerda e direita; será considerada a média entre as duas medidas. As imagens serão obtidas e analisadas seguindo-se as recomendações do “Consensus Statement from the American Society of Echocardiography”^26^ e do “Mannhein Carotid Intima-Media Thickness Concensus (2004-2006)^27^.

Será feita também a pesquisa de placas de aterosclerose, e quando de sua presença, serão classificadas quanto à ecogenicidade, utilizando-se os critérios propostos por Gray-Weale^28^

- Tipo I: placas hipoecoicas;
- Tipo II: placas predominantemente hipoecoidas;
- Tipo III: placas predominantemente hiperecoicas;
- Tipo IV: placas hiperecoicas;
- Tipo V: placa calcificada com sombra acústica.

Quanto mais hipoecoicas (tipo I) forem as placas maior seria sua relação com eventos cardiovasculares.

**Avaliação da qualidade de vida**

Será solicitado aos pacientes, o preenchimento de um questionário sobre a qualidade de vida relacionada à saúde, o *Medical Outcomes Study 36-item Short-Form Health Survey* (ANEXO 3), para verificar o impacto do programa de EF sobre este aspecto^29^.

**Avaliação dos biomarcadores metabólicos**

As amostras de sangue deverão ser colhidas com anticoagulantes obtidas após jejum noturno de 12 horas, por meio de punção venosa em sistema fechado a vácuo Vacutainer® (Becton Dickinson)^30,31^.

- **Glicose plasmática**: A glicose será quantificada por meio da utilização do “kit” comercial da Johnson – EUA**.**
- **Triacilglicerol**: O triacilglicerol será analisado pelo método enzimático colorimétrico
- **Colesterol Total**: O colesterol total será analisado pelo método enzimático calorimétrico.
- **HDL-Colesterol**: O HDL-colesterol será quantificado por um método de precipitação (Lopes-Virella et al, 1977).
- **LDL-Colesterol**: O cálculo do LDL-colesterol será realizado pela formula de Friedewald para triacilglicerol abaixo de 400,0 mg/dL. (NCEP - ATP III 2001) ^31^.
- ***LDL-colesterol = Colesterol Total − HDL-colestero − Trigliçerides***

***5***

- **Ácido Úrico**: O ácido úrico será quantificado pelo método enzimático calorimétrico
- **Hemograma**: O hemograma será realizado por sistema automatizado utilizando-se citometria de fluxo.

**Avaliação dos biomarcadores Inflamatórios e BNP**

A coleta de sangue, para dosagem dos BNP no plasma, será feita por punção venosa, onde uma amostra de 10 mL de sangue venoso será coletada diretamente em tubo do sistema Vacutainer® (Becton Dickinson), contendo heparina e devidamente identificado. A amostra de sangue será imediatamente centrifugada durante 10 minutos a 2000 rpm e na temperatura ambiente, para obtenção de plasma, cujo volume será aliquotado e armazenado à -80ºC até o momento da determinação das concentrações dos biomarcadores.

A determinação do BNP no plasma será realizada empregando-se ensaio imunoenzimático competitivo (ELISA), realizado de acordo com as instruções do fabricante (Wuhan EIAab, Science Co., Ltd, China, cat no. E0541h). Inicialmente 50 uL da solução padrão de BNP ou da amostra de plasma será adicionada aos orifícios da placa. A solução de detecção A (50 uL) será imediatamente adicionada aos orifícios e a placa incubada por 60 min a 37^o^C. A seguir a placa será submetida à lavagem por quatro vezes com solução tampão e em seguida a reação será incubada com 100 uL do reagente revelador por 45 min a 37^o^C. Após novo ciclo de lavagens da placa, 90 uL da solução de substrato serão adicionados a cada orifício e a placa será incubada à temperatura ambiente e ao abrigo da luz por 20 min. A reação será bloqueada pela adição de 50μL de ácido sulfúrico 2M e a leitura da placa será realizada em leitor de ELISA (Multiskan EFLAB, Helsinki, Finland) com comprimento de onda de 450 nm. O limite de sensibilidade do kit será de 3,90 pg/mL.

**Determinação das citocinas TNF-α, IL-1 β, IL-6, IL-10 e da Proteína C-reativa**

Para quantificação das citocinas e da Proteína C-reativa serão empregados kits comerciais Quantikine ELISA (R&D Systems, Minneapolis, MN, USA). As reações serão desenvolvidas segundo as instruções do fabricante e descritas conforme a técnica abaixo.

A placas de 96 orifícios e fundo plano (MaxiSorp-Nunc Life Tech. Inc., Maryland, MA, USA) previamente sensibilizadas com anticorpo monoclonal anti-citocina específica ou anti-PCR receberão 200μL do plasma ou das citocinas recombinantes (R&D Systems). Após 2h de incubação à temperatura ambiente a placa será submetida a lavagem por quatro vezes com solução tampão e em seguida incubada com 200 uL de anticorpo revelador policlonal anti-citocina ou anti-PCR (R&D Systems), seguido de incubação por 2h à temperatura ambiente. A placa sera lavada novamente e adicionados 100μL de estreptoavidina conjugada com peroxidase (R&D Systems), na concentração de 2μg/mL por 20 min a 37ºC, seguido pela lavagem da placa com PBST. Após esse período, serão adicionados 100μL do substrato enzimático, constituído por soluções estabilizadoras de peróxido de hidrogênio e de tetrametilbenzidina (DY999 – R&D Systems). As placas serão incubadas à temperatura ambiente ao abrigo da luz, por 20min e a reação será bloqueada pela adição de 50μL de ácido sulfúrico 2M. A leitura da placa será realizada em leitor de ELISA (Multiskan EFLAB, Helsinki, Finland) com comprimento de onda de 450nm. As concentrações das citocinas presentes nos sobrenadantes de cultura dos monócitos, tratados ou não com LPS e PGN, serão calculadas a partir das curvas-padrão realizada com as diferentes citocinas recombinantes humanas. Nos ensaios, as concentrações dos anticorpos monoclonais e policlonais, bem como das citocinas recombinantes específicas, utilizadas nas curvas-padrão, serão as recomendadas pelo fabricante (R&D Systems). O limite de sensibilidade dos kits será de 1,6 pg/mL para TNF-α, 3,9 pg/mL para IL-1 β e IL-10, de 1pg/mL para IL-6 e de 0,010 ng/mL para PCR.

**5 – PROTOCOLO DE EXERCÍCIO**

O programa de exercício terá duração de 4 meses, e durante este período os indivíduos serão orientados a praticar as atividades pelo menos 3 vezes por semana, não ultrapassando 5 vezes semanais, sendo pelo menos uma das atividades supervisionada.

O protocolo de exercícios terá como base atividades aeróbias de baixa intensidade com duração crescente a medida que os indivíduos apresentem melhora do condicionamento físico.

O protocolo de exercício terá três momentos:

**Fase inicial:** Serão realizados exercícios calistênicos, sugeridos durante os dias de aula presencial (atividades supervisionadas), sendo que esses exercícios terão duração de aproximadamente 10 minutos com o objetivo de aquecer a musculatura a ser trabalhada.

**Parte principal:** O protocolo de exercício contara inicialmente com uma caminhada de 35 minutos ininterruptas com intensidade entre 60 a 75 % da frequência cardíaca máxima determinada pelo teste ergométrico. Com a melhora do condicionamento físico o tempo de caminhada deverá ser aumentado gradualmente até atingir 50 minutos de caminhada.

**Parte final:** Será destinada ao alongamento e relaxamento dos grupos musculares trabalhados, sendo que essa atividades também serão orientadas nos dias das aulas supervisionadas .

Esse protocolo de exercício preconiza atividades de baixa intensidade e longa duração para indivíduos que apresentam doença falciforme, pois exercícios de intensidades altas podem desencadear crises dolorosas e não apresentam evidências de melhora das variáveis cardiovasculares em indivíduos com doença arterial coronariana. Outro ponto relevante deste protocolo é a importância das atividades de alongamento e relaxamento, pois está relacionado com melhora na qualidade de vida e da dor muscular em indivíduos normais após a atividade física.

**6 – ANÁLISE ESTATÍSTICA**

As variáveis contínuas serão apresentadas como médias e desvios padrão ou medianas e intervalos interquartílicos. As variáveis categóricas serão apresentadas como proporções. As comparações entre grupos serão efetuadas por meio do teste “t” de Student ou Mann-Whitney ou teste do quiquadrado. As comparações entre os três momentos do protocolo de estudo serão efetuadas por meio de ANOVA para medidas repetidas. As associações entre as variáveis clínicas e laboratoriais e o efeito da prescrição do exercício físico serão avaliadas por meio de modelos de regressão linear ou logística ou Coeficiente de Correlação de Spearman. Em todos os casos será adotado o nível de significância p<0,05.

**Análise crítica dos possíveis riscos e benefícios**

A pesquisa em si não acarreta nenhum risco para os pacientes do grupo controle, visto que seu tratamento quanto às possíveis comorbidades será realizado de acordo com os consensos já estabelecidos na literatura e as avaliações e exames de rotina a que serão submetidos não oferecem nenhum risco adicional. Nenhuma droga será adicionada ou retirada para o propósito da pesquisa. Para os pacientes do grupo intervenção (AFEXE), não se espera risco iminente inerente à prática de EF, devido aos cuidados na criação do protocolo de treinamento e constante acompanhamento do profissional de Educação Física habilitado (Jonas Alves de Araujo Junior) nas sessões de exame físico e de médico cardiologista e hematologista responsável (Meliza Goi Roscani e Newton Key Hokama). Os pacientes receberão hidratação adequada e monitorização dos sinais vitais Contudo, alguns desconfortos, como sudorese aumentada durante a prática de EF e dor muscular aguda característica da fase inicial de treinamento, podem ocorrer em decorrência da participação no programa de EF. No entanto, em caso de sintomas sugestivos de dor articular, falta de ar, cansaço limitante, o exercício físico será interrompido imediatamente. De acordo com a gracidade dos sintomas apresentados, o paciente será excluído do protocolo de exercício.

**7 – ÉTICA**

Os pacientes serão selecionados e convidados a participar da pesquisa, respeitando-se sempre o termo de consentimento livre e esclarecido e as normas do comitê de ética. Serão submetidos à avaliação clínica e à metodologia descrita previamente, após o aceite e a assinatura do Termo de Consentimento Livre e Esclarecido (anexo 3). Os dados anotados na ficha clínica em anexo serão analisados conforme o tratamento estatístico já descrito e publicados, preservando-se a identidade e a dignidade dos pacientes, de acordo com as resoluções 196/96 e 251/97 do Conselho Nacional de Saúde.

Todos os pacientes estáveis, seja do grupo controle ou do grupo intervenção, após o término da pesquisa, se houver benefícios, serão estimulados à prática regular de EF adequados e encaminhados, por intermédio de um relatório, para redes de academias de ginástica ou locais apropriados para a prática de EF com profissionais habilitados.
